# Supplementary material for: Diagnostic efficiency and validity of the DSM-oriented Child Behavior Checklist and Youth Self-Report scales in a clinical sample of Swedish youth
Source: PLoS One. 2021 Jul 22;16(7):e0254953. doi: 10.1371/journal.pone.0254953 (PMC8297893; doi:10.1371/journal.pone.0254953)
Supplement: S1 File — (DOCX) [file pone.0254953.s001.docx]

Diagnostic efficiency and validity of the DSM-oriented Child Behavior Checklist and Youth Self-Report scales in a clinical sample of Swedish youth

S1 File

Supplemental tables 1-12

Receiver operating characteristics results for each incremental score on the CBCL and YSR versus a LEAD diagnosis.

S1 Table. Receiver operating characteristics results for each incremental score on the CBCL and YSR versus a LEAD diagnosis: CBCL Affective -> Any depression

| Cutoff Score | Sensitivity  (95% CI) | Specificity  (95% CI) | Kappa | Level | DLR+  (95% CI) | DLR-  (95% CI) | PPV  (95% CI) | NPV  (95% CI) |
| --- | --- | --- | --- | --- | --- | --- | --- | --- |
| ≥3 | 94.81  87.2 - 98.6 | 37.10  30.1 - 44.5 | .223 | .722 | 1.51  1.3 - 1.7 | 0.14  0.05 - 0.4 | 38.4  35.6 - 41.3 | 94.5  86.7 - 97.9 |
| ≥4 | 89.61  80.6 - 95.4 | 45.70  38.4 - 53.1 | .261 | .646 | 1.65  1.4 - 1.9 | 0.23  0.1 - 0.4 | 40.6  37.0 - 44.3 | 91.4  84.4 - 95.4 |
| ≥5 | 87.01  77.4 - 93.6 | 51.61  44.2 - 59.0 | .296 | .597 | 1.80  1.5 - 2.1 | 0.25  0.1 - 0.5 | 42.7  38.5 - 46.9 | 90.6  84.1 - 94.6 |
| ≥6 | 79.22  68.5 - 87.6 | 58.06  50.6 - 65.2 | .302 | .529 | 1.89  1.5 - 2.3 | 0.36  0.2 - 0.6 | 43.9  38.9 - 49.0 | 87.1  81.1 - 91.4 |
| ≥7 | 75.32  64.2 - 84.4 | 69.89  62.8 - 76.4 | .396 | .433 | 2.50  1.9 - 3.2 | 0.35  0.2 - 0.5 | 50.9  44.6 - 57.2 | 87.2  82.1 - 91.1 |
| ≥8 | 57.14  45.4 - 68.4 | 77.42  70.7 - 83.2 | .334 | .327 | 2.53  1.8 - 3.5 | 0.55  0.4 - 0.7 | 51.2  43.0 - 59.3 | 81.4  76.9 - 85.1 |
| ≥9 | 48.05  36.5 - 59.7 | 86.02  80.2 - 90.7 | .360 | .240 | 3.44  2.2 - 5.3 | 0.60  0.5 - 0.8 | 58.7  48.2 - 68.5 | 80.0  76.2 - 83.3 |
| ≥10 | 35.06  24.5 - 46.8 | 90.86  85.8 - 94.6 | .296 | .167 | 3.84  2.2 - 6.6 | 0.71  0.6 - 0.8 | 61.4  47.9 - 73.3 | 77.2  74.0 - 80.0 |

Level = Test positive rate (percentage of cases scoring positive)

DLR = Diagnostic likelihood ratio

DLR+ = Ratio between the probability of a positive test result given the presence of the disease and the probability of a positive test result given the absence of the disease

DLR- = Ratio between the probability of a negative test result given the presence of the disease and the probability of a negative test result given the absence of the disease

PPV= Positive predictive value (probability that the disease is present when the test is positive).

NPV = Negative predictive value (probability that the disease is not present when the test is negative).

S2 Table. Receiver operating characteristics results for each incremental score on the CBCL and YSR versus a LEAD diagnosis: CBCL Anxiety –> Any anxiety

| Cutoff Score | Sensitivity  (95% CI) | Specificity  (95% CI) | Kappa | Level | DLR+  (95% CI) | DLR-  (95% CI) | PPV  (95% CI) | NPV  (95% CI) |
| --- | --- | --- | --- | --- | --- | --- | --- | --- |
| ≥2 | 91.58  84.1 - 96.3 | 36.90  29.6 - 44.7 | .233 | .734 | 1.45  1.3 - 1.7 | 0.23  0.1 - 0.5 | 45.1  41.9 - 48.3 | 88.6  79.5 - 93.9 |
| ≥3 | 85.26  76.5 - 91.7 | 48.21  40.5 - 56.0 | .287 | .639 | 1.65  1.4 - 1.9 | 0.31  0.2 - 0.5 | 48.2  44.0 - 52.4 | 85.3  77.7 - 90.6 |
| ≥4 | 73.68  63.6 - 82.2 | 63.69  55.9 - 71.0 | .345 | .498 | 2.03  1.6 - 2.6 | 0.41  0.3 - 0.6 | 53.4  47.6 - 59.2 | 81.1  75.0 - 85.9 |
| ≥5 | 58.95  48.4 - 68.9 | 75.00  67.7 - 81.3 | .337 | .373 | 2.36  1.7 - 3.2 | 0.55  0.4 - 0.7 | 57.1  49.4 - 64.5 | 76.4  71.4 - 80.7 |
| ≥6 | 51.58  41.1 - 62.0 | 82.14  75.5 - 87.6 | .350 | .300 | 2.89  2.0 - 4.2 | 0.59  0.5 - 0.7 | 62.0  52.8 - 70.5 | 75.0  70.7 - 78.9 |
| ≥7 | 37.89  28.1 - 48.4 | 89.29  83.6 - 93.5 | .300 | .205 | 3.54  2.1 - 5.9 | 0.70  0.6 - 0.8 | 66.7  54.6 - 76.9 | 71.8  68.3 - 75.0 |
| ≥8 | 30.53  21.5 - 40.8 | 92.26  87.1 - 95.8 | .259 | .160 | 3.94  2.2 - 7.2 | 0.75  0.7 - 0.9 | 69.0  54.9 - 80.3 | 70.1  67.1 - 73.0 |

Level = Test positive rate (percentage of cases scoring positive)

DLR = Diagnostic likelihood ratio

DLR+ = Ratio between the probability of a positive test result given the presence of the disease and the probability of a positive test result given the absence of the disease

DLR- = Ratio between the probability of a negative test result given the presence of the disease and the probability of a negative test result given the absence of the disease

PPV= Positive predictive value (probability that the disease is present when the test is positive).

NPV = Negative predictive value (probability that the disease is not present when the test is negative).

S3 Table. Receiver operating characteristics results for each incremental score on the CBCL and YSR versus a LEAD diagnosis: CBCL ADHD -> ADHD

| Cutoff Score | Sensitivity  (95% CI) | Specificity  (95% CI) | Kappa | Level | DLR+  (95% CI) | DLR-  (95% CI) | PPV  (95% CI) | NPV  (95% CI) |
| --- | --- | --- | --- | --- | --- | --- | --- | --- |
| ≥4 | 89.21 (82.8 - 93.8) | 46.77  (37.8 - 55.9) | .368 | .722 | 1.68  (1.4 - 2.0) | 0.23  (0.1 - 0.4) | 65.3  (61.2 - 69.1) | 79.5  (69.8 - 86.6) |
| ≥5 | 85.61  (78.7 - 91.0) | 57.26  (48.1 - 66.1) | .435 | .654 | 2.00  (1.6 - 2.5) | 0.25  (0.2 - 0.4) | 69.2  (64.4 - 73.6) | 78.0  (69.7 - 84.6) |
| ≥6 | 80.58  (73.0 - 86.8) | 70.16  (61.3 - 78.0) | .510 | .567 | 2.70  (2.0 - 3.6) | 0.28  (0.2 - 0.4) | 75.2  (69.5 - 80.1) | 76.3  (69.3 - 82.2) |
| ≥7 | 69.78  (61.4 - 77.3) | 77.42  (69.0 - 84.4) | .469 | .475 | 3.09  (2.2 - 4.4) | 0.39  (0.3 - 0.5) | 77.6  (71.1 - 83.0) | 69.6  (63.6 - 75.0) |
| ≥8 | 61.87  (53.3 - 70.0) | 83.06  (75.3 - 89.2) | .443 | .407 | 3.65  (2.4 - 5.5) | 0.46  (0.4 - 0.6) | 80.4  (73.1 - 86.1) | 66.0  (60.8 - 70.9) |
| ≥9 | 46.76  (38.3 - 55.4) | 90.32  (83.7 - 94.9) | .361 | .293 | 4.83  (2.7 - 8.5) | 0.59  (0.5 - 0.7) | 84.4  (75.5 - 90.5) | 60.2  (56.2 - 64.1) |

Level = Test positive rate (percentage of cases scoring positive)

DLR = Diagnostic likelihood ratio

DLR+ = Ratio between the probability of a positive test result given the presence of the disease and the probability of a positive test result given the absence of the disease

DLR- = Ratio between the probability of a negative test result given the presence of the disease and the probability of a negative test result given the absence of the disease

PPV= Positive predictive value (probability that the disease is present when the test is positive).

NPV = Negative predictive value (probability that the disease is not present when the test is negative).

S4 Table. Receiver operating characteristics results for each incremental score on the CBCL and YSR versus a LEAD diagnosis: CBCL ODD -> ODD

| Cutoff Score | Sensitivity  (95% CI) | Specificity  (95% CI) | Kappa | Level | DLR+  (95% CI) | DLR-  (95% CI) | PPV  (95% CI) | NPV  (95% CI) |
| --- | --- | --- | --- | --- | --- | --- | --- | --- |
| ≥4 | 95.08  (86.3 - 99.0) | 46.04  (39.0 - 53.2) | .256 | .635 | 1.76  (1.5 - 2.0) | 0.11  (0.04 - 0.3) | 34.7  (31.6 - 38.0) | 96.9  (91.1 - 99.0) |
| ≥5 | 86.89  (75.8 - 94.2) | 55.45  (48.3 - 62.4) | .288 | .544 | 1.95  (1.6 - 2.3) | 0.24  (0.1 - 0.5) | 37.1  (32.9 - 41.4) | 93.3  (87.9 - 96.4) |
| ≥6 | 81.97  (70.0 - 90.6) | 68.32  (61.4 - 74.7) | .386 | .433 | 2.59  (2.0 - 3.3) | 0.26  (0.2 - 0.5) | 43.9  (38.2 - 49.7) | 92.6  (87.9 - 95.6) |
| ≥7 | 63.93  (50.6 - 75.8) | 76.24  (69.8 - 81.9) | .350 | .331 | 2.69  (2.0 - 3.7) | 0.47  (0.3 - 0.7) | 44.8  (37.3 - 52.6) | 87.5  (83.2 - 90.8) |
| ≥8 | 52.46  (39.3 - 65.4) | 87.13  (81.7 - 91.4) | .403 | .221 | 4.08  (2.6 - 6.3) | 0.55  (0.4 - 0.7) | 55.2  (44.4 - 65.4) | 85.9  (82.3 - 88.8) |
| ≥9 | 24.59  (14.5 - 37.3) | 96.04  (92.3 - 98.3) | .264 | .087 | 6.21  (2.8 - 13.9) | 0.79  (0.7 - 0.9) | 65.2  (45.5 - 80.8) | 80.8  (78.5 - 83.0) |

Level = Test positive rate (percentage of cases scoring positive)

DLR = Diagnostic likelihood ratio

DLR+ = Ratio between the probability of a positive test result given the presence of the disease and the probability of a positive test result given the absence of the disease

DLR- = Ratio between the probability of a negative test result given the presence of the disease and the probability of a negative test result given the absence of the disease

PPV= Positive predictive value (probability that the disease is present when the test is positive).

NPV = Negative predictive value (probability that the disease is not present when the test is negative).

S5 Table. Receiver operating characteristics results for each incremental score on the CBCL and YSR versus a LEAD diagnosis: CBCL CD -> CD

| Cutoff Score | Sensitivity  (95% CI) | Specificity  (95% CI) | Kappa | Level | DLR+  (95% CI) | DLR-  (95% CI) | PPV  (95% CI) | NPV  (95% CI) |
| --- | --- | --- | --- | --- | --- | --- | --- | --- |
| ≥10 | 90.91  (58.7 - 99.8) | 82.14  (76.8 - 86.7) | .251 | .209 | 5.09  (3.7 - 7.0) | 0.11  (0.02 - 0.7) | 18.2  (13.8 - 23.5) | 99.5  (97.0 - 99.9) |
| ≥11 | 81.82  (48.2 - 97.7) | 85.71  (80.8 - 89.8) | .273 | .171 | 5.73  (3.8 - 8.6) | 0.21  (0.06 - 0.7) | 20.0  (14.2 - 27.4) | 99.1  (96.9 - 99.7) |
| ≥12 | 63.64  (30.8 - 89.1) | 89.68  (85.2 - 93.1) | .273 | .125 | 6.17  (3.5 - 11.0) | 0.41  (0.2 - 0.9) | 21.2  (13.1 - 32.4) | 98.3  (96.3 - 99.2) |
| ≥13 | 63.64  (30.8 - 89.1) | 92.86  (88.9 - 95.7) | .351 | .095 | 8.91  (4.7 - 16.7) | 0.39  (0.2 - 0.9) | 28.0  (17.1 - 42.2) | 98.3  (96.4 - 99.2) |
| ≥14 | 54.55  (23.4 - 83.3) | 95.24  (91.8 - 97.5) | .382 | .068 | 11.45  (5.3 - 24.8) | 0.48  (0.2 - 0.9) | 33.3  (18.8 - 52.0) | 98.0  (96.2 - 98.9) |

Level = Test positive rate (percentage of cases scoring positive)

DLR = Diagnostic likelihood ratio

DLR+ = Ratio between the probability of a positive test result given the presence of the disease and the probability of a positive test result given the absence of the disease

DLR- = Ratio between the probability of a negative test result given the presence of the disease and the probability of a negative test result given the absence of the disease

PPV= Positive predictive value (probability that the disease is present when the test is positive).

NPV = Negative predictive value (probability that the disease is not present when the test is negative).

S6 Table. Receiver operating characteristics results for each incremental score on the CBCL and YSR versus a LEAD diagnosis: CBCL OCD -> OCD

| Cutoff Score | Sensitivity  (95% CI) | Specificity  (95% CI) | Kappa | Level | DLR+  (95% CI) | DLR-  (95% CI) | PPV  (95% CI) | NPV  (95% CI) |
| --- | --- | --- | --- | --- | --- | --- | --- | --- |
| ≥3 | 83.33  (51.6 - 97.9) | 56.18  (49.8 - 62.4) | .075 | .456 | 1.90  (1.4 - 2.5) | 0.30  (0.08 - 1.1) | 8.3  (6.4 - 10.8) | 98.6  (95.2 - 99.6) |
| ≥4 | 83.33  (51.6 - 97.9) | 64.94  (58.7 - 70.8) | .109 | .373 | 2.38  (1.8 - 3.2) | 0.26  (0.07 - 0.9) | 10.2  (7.7 - 13.3) | 98.8  (95.8 - 99.7) |
| ≥5 | 83.33  (51.6 - 97.9) | 76.49  (70.8 - 81.6) | .183 | .262 | 3.55  (2.5 - 5.0) | 0.22  (0.06 - 0.8) | 14.5  (10.8 - 19.2) | 99.0  (96.4 - 99.7) |
| ≥6 | 83.33  (51.6 - 97.9) | 85.66  (80.7 - 89.7) | .294 | .175 | 5.81  ((3.9 - 8.6) | 0.19  (0.05 - 0.7) | 21.7  (15.8 - 29.2) | 99.1  (96.8 - 99.7) |
| ≥7 | 83.33  (51.6 - 97.9) | 91.63  (87.5 - 94.7) | .427 | .118 | 9.96  (6.2 - 16.1) | 0.18  (0.05 - 0.6) | 32.3  (22.7 - 43.5) | 99.1  (97.0 - 99.8) |
| ≥8 | 75.00  (42.8 - 94.5) | 94.42  (90.8 - 96.9) | .483 | .087 | 13.45  (7.3 - 24.6) | 0.26  (0.10 - 0.7) | 39.1  (26.0 - 54.1) | 98.7  (96.7 - 99.5) |

Level = Test positive rate (percentage of cases scoring positive)

DLR = Diagnostic likelihood ratio

DLR+ = Ratio between the probability of a positive test result given the presence of the disease and the probability of a positive test result given the absence of the disease

DLR- = Ratio between the probability of a negative test result given the presence of the disease and the probability of a negative test result given the absence of the disease

PPV= Positive predictive value (probability that the disease is present when the test is positive).

NPV = Negative predictive value (probability that the disease is not present when the test is negative).

S7 Table. Receiver operating characteristics results for each incremental score on the CBCL and YSR versus a LEAD diagnosis: YSR Affective -> Any depression

| Cutoff Score | Sensitivity  (95% CI) | Specificity  (95% CI) | Kappa | Level | DLR+  (95% CI) | DLR-  (95% CI) | PPV  (95% CI) | NPV  (95% CI) |
| --- | --- | --- | --- | --- | --- | --- | --- | --- |
| ≥3 | 90.16  (79.8 - 96.3) | 20.51  (12.2 - 31.2) | .100 | .842 | 1.13  (1.0 - 1.3) | 0.48  (0.2 - 1.2) | 47.0  (43.5 - 50.5) | 72.7  (52.6 - 86.5) |
| ≥4 | 85.25  (73.8 - 93.0) | 26.92  (17.5 - 38.2) | .112 | .784 | 1.17  (1.0 - 1.4) | 0.55  (0.3 - 1.1) | 47.7  (43.5 - 52.0) | 70.0  (53.5 - 82.5) |
| ≥5 | 83.61  (71.9 - 91.8) | 43.59  (32.4 - 55.3) | .256 | .683 | 1.48  (1.2 - 1.9) | 0.38  (0.2 - 0.7) | 53.7  (48.1 - 59.2) | 77.3  (64.6 - 86.3) |
| ≥6 | 78.69  (66.3 - 88.1) | 60.26  (48.5 - 71.2) | .377 | .568 | 1.98  (1.5 - 2.7) | 0.35  (0.2 - 0.6) | 60.8  (53.4 - 67.7) | 78.3  (68.4 - 85.8) |
| ≥7 | 73.77  (60.9 - 84.2) | 66.67  (55.1 - 76.9) | .397 | .511 | 2.21  (1.6 - 3.1) | 0.39  (0.3 - 0.6) | 63.4  (55.0 - 71.0) | 76.5  (67.5 - 83.6) |
| ≥8 | 68.85  (55.7 - 80.1) | 73.08  (61.8 - 82.5) | .418 | .453 | 2.56  (1.7 - 3.8) | 0.43  (0.3 - 0.6) | 66.7  (57.2 - 74.9) | 75.0  (66.9 - 81.7) |
| ≥9 | 67.21  (54.0 - 78.7) | 75.64  (64.6 - 84.7) | .429 | .432 | 2.76  (1.8 - 4.2) | 0.43  (0.3 - 0.6) | 68.3  (58.4 - 76.8) | 74.7  (66.8 - 81.2) |
| ≥10 | 57.38  (44.1 - 70.0) | 80.77  (70.3 - 88.8) | .389 | .360 | 2.98  (1.8 - 4.9) | 0.53  (0.4 - 0.7) | 70.0  (58.5 - 79.4) | 70.8  (64.0 - 76.8) |
| ≥11 | 52.46  (39.3 - 65.4) | 87.18  (77.7 - 93.7) | .410 | .302 | 4.09  (2.2 - 7.7) | 0.55  (0.4 - 0.7) | 76.2  (63.1 - 85.7) | 70.1  (64.0 - 75.6) |
| ≥12 | 39.34  (27.1 - 52.7) | 91.03  (82.4 - 96.3) | .321 | .223 | 4.38  (2.0 - 9.5) | 0.67  (0.5 - 0.8) | 77.4  (61.3 - 88.1) | 65.7  (60.8 - 70.4) |

Level = Test positive rate (percentage of cases scoring positive)

DLR = Diagnostic likelihood ratio

DLR+ = Ratio between the probability of a positive test result given the presence of the disease and the probability of a positive test result given the absence of the disease

DLR- = Ratio between the probability of a negative test result given the presence of the disease and the probability of a negative test result given the absence of the disease

PPV= Positive predictive value (probability that the disease is present when the test is positive).

NPV = Negative predictive value (probability that the disease is not present when the test is negative).

S8 Table. Receiver operating characteristics results for each incremental score on the CBCL and YSR versus a LEAD diagnosis: YSR Anxiety –> Any anxiety

| Cutoff Score | Sensitivity  (95% CI) | Specificity  (95% CI) | Kappa | Level | DLR+  (95% CI) | DLR-  (95% CI) | PPV  (95% CI) | NPV  (95% CI) |
| --- | --- | --- | --- | --- | --- | --- | --- | --- |
| ≥2 | 93.75  82.8 - 98.7 | 21.98  14.0 - 31.9 | .117 | .835 | 1.20  1.1 - 1.4 | 0.28  0.09 - 0.9 | 38.8  35.7 - 42.0 | 87.0  67.6 - 95.5 |
| ≥3 | 83.33  69.8 - 92.5 | 43.96  33.6 - 54.8 | .225 | .655 | 1.49  1.2 - 1.9 | 0.38  0.2 - 0.7 | 44.0  38.6 - 49.5 | 83.3  71.8 - 90.7 |
| ≥4 | 75.00  60.4 - 86.4 | 56.04  45.2 - 66.4 | .273 | .547 | 1.71  1.3 - 2.3 | 0.45  0.3 - 0.8 | 47.4  40.4 - 54.4 | 81.0  71.6 - 87.8 |
| ≥5 | 64.58  49.5 - 77.8 | 68.13  57.5 - 77.5 | .309 | .432 | 2.03  1.4 - 2.9 | 0.52  0.3 - 0.8 | 51.7  42.6 - 60.7 | 78.5  70.8 - 84.6 |
| ≥6 | 60.42  45.3 - 74.2 | 74.73  64.5 - 83.3 | .345 | .374 | 2.39  1.6 - 3.6 | 0.53  0.4 - 0.8 | 55.8  45.3 - 65.8 | 78.2  71.2 - 83.8 |
| ≥7 | 41.67  27.6 - 56.8 | 83.52  74.3 - 90.5 | .269 | .252 | 2.53  1.4 - 4.5 | 0.70  0.5 - 0.9 | 57.1  43.0 - 70.2 | 73.1  67.8 - 77.8 |
| ≥8 | 27.08  15.3 - 41.8 | 87.91  79.4 - 93.8 | .170 | .173 | 2.24  1.1 - 4.6 | 0.83  0.7 - 1.0 | 54.2  36.5 - 70.9 | 69.6  65.4 - 73.4 |
| ≥9 | 25.00  13.6 - 39.6 | 94.51  87.6 - 98.2 | .230 | .122 | 4.55  1.7 - 12.2 | 0.79  0.7 - 0.9 | 70.6  47.3 - 86.5 | 70.5  66.8 - 73.9 |

Level = Test positive rate (percentage of cases scoring positive)

DLR = Diagnostic likelihood ratio

DLR+ = Ratio between the probability of a positive test result given the presence of the disease and the probability of a positive test result given the absence of the disease

DLR- = Ratio between the probability of a negative test result given the presence of the disease and the probability of a negative test result given the absence of the disease

PPV= Positive predictive value (probability that the disease is present when the test is positive).

NPV = Negative predictive value (probability that the disease is not present when the test is negative).

S9 Table. Receiver operating characteristics results for each incremental score on the CBCL and YSR versus a LEAD diagnosis: YSR ADHD -> ADHD

| Cutoff Score | Sensitivity  (95% CI) | Specificity  (95% CI) | Kappa | Level | DLR+  (95% CI) | DLR-  (95% CI) | PPV  (95% CI) | NPV  (95% CI) |
| --- | --- | --- | --- | --- | --- | --- | --- | --- |
| ≥3 | 93.33  83.8 - 98.2 | 18.99  11.0 - 29.4 | .110 | .863 | 1.15  1.0 - 1.3 | 0.35  0.1 - 1.0 | 46.7  43.5 - 49.8 | 78.9  56.7 - 91.5 |
| ≥4 | 85.00  73.4 - 92.9 | 32.91  22.7 - 44.4 | .165 | .748 | 1.27  1.1 - 1.5 | 0.46  0.2 - 0.9 | 49.0  44.4 - 53.7 | 74.3  59.4 - 85.1 |
| ≥5 | 81.67  69.6 - 90.5 | 44.30  33.1 - 55.9 | .244 | .669 | 1.47  1.2 - 1.8 | 0.41  0.2 - 0.7 | 52.7  46.9 - 58.4 | 76.1  63.9 - 85.1 |
| ≥6 | 75.00  62.1 - 85.3 | 53.16  41.6 - 64.5 | .270 | .590 | 1.60  1.2 - 2.1 | 0.47  0.3 - 0.8 | 54.9  48.0 - 61.6 | 73.7  63.3 - 82.0 |
| ≥7 | 65.00  51.6 - 76.9 | 69.62  58.2 - 79.5 | .344 | .453 | 2.14  1.5 - 3.1 | 0.50  0.3 - 0.7 | 61.9  52.6 - 70.4 | 72.4  64.3 - 79.2 |
| ≥8 | 48.33  35.2 - 61.6 | 82.28  72.1 - 90.0 | .317 | .309 | 2.73  1.6 - 4.7 | 0.63  0.5 - 0.8 | 67.4  54.6 - 78.1 | 67.7  61.7 - 73.2 |
| ≥9 | 38.33  26.1 - 51.8 | 87.34  78.0 - 93.8 | .271 | .237 | 3.03  1.6 - 5.9 | 0.71  0.6 - 0.9 | 69.7  54.3 - 81.7 | 65.1  60.0 - 69.8 |
| ≥10 | 20.00  10.8 - 32.3 | 93.67  85.8 - 97.9 | .150 | .122 | 3.16  1.2 - 8.5 | 0.85  0.7 - 1.0 | 70.6  47.2 - 86.6 | 60.7  57.3 - 63.9 |

Level = Test positive rate (percentage of cases scoring positive)

DLR = Diagnostic likelihood ratio

DLR+ = Ratio between the probability of a positive test result given the presence of the disease and the probability of a positive test result given the absence of the disease

DLR- = Ratio between the probability of a negative test result given the presence of the disease and the probability of a negative test result given the absence of the disease

PPV= Positive predictive value (probability that the disease is present when the test is positive).

NPV = Negative predictive value (probability that the disease is not present when the test is negative).

S10 Table. Receiver operating characteristics results for each incremental score on the CBCL and YSR versus a LEAD diagnosis: YSR ODD -> ODD

| Cutoff Score | Sensitivity  (95% CI) | Specificity  (95% CI) | Kappa | Level | DLR+  (95% CI) | DLR-  (95% CI) | PPV  (95% CI) | NPV  (95% CI) |
| --- | --- | --- | --- | --- | --- | --- | --- | --- |
| ≥3 | 96.15  80.4 - 99.9 | 18.58  11.9 - 27.0 | .063 | .842 | 1.18  1.1 - 1.3 | 0.21  0.03 - 1.5 | 21.4  19.5 - 23.4 | 95.5  74.7 - 99.3 |
| ≥4 | 80.77  60.6 - 93.4 | 30.09  21.8 - 39.4 | .052 | .719 | 1.16  0.9 - 1.4 | 0.64  0.3 - 1.5 | 21.0  17.5 - 24.9 | 87.2  74.7 - 94.0 |
| ≥5 | 69.23  48.2 - 85.7 | 47.79  38.3 - 57.4 | .097 | .554 | 1.33  1.0 - 1.8 | 0.64  0.4 - 1.2 | 23.4  18.3 - 29.4 | 87.1  78.6 - 92.5 |
| ≥6 | 53.85  33.4 - 73.4 | 69.91  60.6 - 78.2 | .179 | .345 | 1.79  1.1 - 2.8 | 0.66  0.4 - 1.0 | 29.2  20.7 - 39.3 | 86.8  81.0 - 91.0 |
| ≥7 | 23.08  9.0 - 43.6 | 80.53  72.0 - 87.4 | .035 | .201 | 1.19  0.5 - 2.6 | 0.96  0.8 - 1.2 | 21.4  11.0 - 37.7 | 82.0  78.3 - 85.1 |
| ≥8 | 3.85  0.10 - 19.6 | 90.27  83.2 - 95.0 | -.074 | .086 | 0.40  0.05 - 2.9 | 1.07  1.0 - 1.2 | 8.3  1.2 - 40.2 | 80.3  78.7 - 81.8 |
| ≥9 | 3.85  0.10 - 19.6 | 92.92  86.5 - 96.9 | -.043 | .065 | 0.54  0.07 - 4.2 | 1.03  0.9 - 1.1 | 11.1  1.6 - 48.9 | 80.8  79.3 - 82.2 |

Level = Test positive rate (percentage of cases scoring positive)

DLR = Diagnostic likelihood ratio

DLR+ = Ratio between the probability of a positive test result given the presence of the disease and the probability of a positive test result given the absence of the disease

DLR- = Ratio between the probability of a negative test result given the presence of the disease and the probability of a negative test result given the absence of the disease

PPV= Positive predictive value (probability that the disease is present when the test is positive).

NPV = Negative predictive value (probability that the disease is not present when the test is negative).

S11 Table. Receiver operating characteristics results for each incremental score on the CBCL and YSR versus a LEAD diagnosis: YSR CD -> CD

| Criterion | Sensitivity  95% CI | Specificity  95% CI | KAppa | Level | +LR  95% CI | -LR  95% CI | +PV  95% CI | -PV  95% CI |
| --- | --- | --- | --- | --- | --- | --- | --- | --- |
| ≥3 | 100.00  63.1 - 100.0 | 33.59  25.6 - 42.4 | .055 | .683 | 1.51  1.3 - 1.7 | 0.00 | 8.4  7.5 - 9.4 | 100.0 |
| ≥4 | 87.50  47.3 - 99.7 | 44.27  35.6 - 53.2 | .061 | .576 | 1.57  1.2 - 2.1 | 0.28  0.04 - 1.8 | 8.8  6.6 - 11.5 | 98.3  90.2 - 99.7 |
| ≥5 | 87.50  47.3 - 99.7 | 59.54  50.6 - 68.0 | .116 | .432 | 2.16  1.5 - 3.0 | 0.21  0.03 - 1.3 | 11.7  8.6 - 15.6 | 98.7  92.5 - 99.8 |
| ≥6 | 87.50  47.3 - 99.7 | 68.70  60.0 - 76.5 | .168 | .345 | 2.80  1.9 - 4.0 | 0.18  0.03 - 1.1 | 14.6  10.6 - 19.7 | 98.9  93.5 - 99.8 |
| ≥7 | 75.00  34.9 - 96.8 | 77.86  69.8 - 84.6 | .205 | .252 | 3.39  2.0 - 5.7 | 0.32  0.10 - 1.1 | 17.1  11.0 - 25.7 | 98.1  93.9 - 99.4 |
| ≥8 | 75.00  34.9 - 96.8 | 80.92  73.1 - 87.3 | .238 | .223 | 3.93  2.3 - 6.7 | 0.31  0.09 - 1.0 | 19.4  12.3 - 29.0 | 98.1  94.1 - 99.4 |
| ≥9 | 75.00  34.9 - 96.8 | 84.73  77.4 - 90.4 | .290 | .187 | 4.91  2.8 - 8.7 | 0.30  0.09 - 1.0 | 23.1  14.5 - 34.6 | 98.2  94.3 - 99.5 |
| ≥10 | 50.00  15.7 - 84.3 | 90.08  83.6 - 94.6 | .262 | .122 | 5.04  2.1 - 12.0 | 0.56  0.3 - 1.1 | 23.5  11.5 - 42.2 | 96.7  93.6 - 98.3 |

Level = Test positive rate (percentage of cases scoring positive)

DLR = Diagnostic likelihood ratio

DLR+ = Ratio between the probability of a positive test result given the presence of the disease and the probability of a positive test result given the absence of the disease

DLR- = Ratio between the probability of a negative test result given the presence of the disease and the probability of a negative test result given the absence of the disease

PPV= Positive predictive value (probability that the disease is present when the test is positive).

NPV = Negative predictive value (probability that the disease is not present when the test is negative).

S12 Table. Receiver operating characteristics results for each incremental score on the CBCL and YSR versus a LEAD diagnosis: YSR OCD -> OCD

| Cutoff Score | Sensitivity  (95% CI) | Specificity  (95% CI) | Kappa | Level | DLR+  (95% CI) | DLR-  (95% CI) | PPV  (95% CI) | NPV  (95% CI) | Cutoff Score |
| --- | --- | --- | --- | --- | --- | --- | --- | --- | --- |
| ≥1 | 100.00  63.1 - 100.0 | 7.63  3.7 - 13.6 | .009 | .928 | 1.08  1.0 - 1.1 | 0.00 | 6.2  5.9 - 6.5 | 100.0 | |
| ≥2 | 87.50  47.3 - 99.7 | 20.61  14.0 - 28.6 | .012 | .799 | 1.10  0.8 - 1.5 | 0.61  0.09 - 3.9 | 6.3  4.9 - 8.1 | 96.4  80.7 - 99.4 | |
| ≥3 | 75.00  34.9 - 96.8 | 35.88  27.7 - 44.7 | .019 | .647 | 1.17  0.8 - 1.8 | 0.70  0.2 - 2.4 | 6.7  4.5 - 9.8 | 95.9  87.4 - 98.8 | |
| ≥4 | 75.00  34.9 - 96.8 | 49.62  40.8 - 58.5 | .052 | .518 | 1.49  1.0 - 2.3 | 0.50  0.1 - 1.7 | 8.3  5.6 - 12.3 | 97.0  90.6 - 99.1 | |
| ≥5 | 62.50  24.5 - 91.5 | 56.49  47.6 - 65.1 | .046 | .446 | 1.44  0.8 - 2.5 | 0.66  0.3 - 1.6 | 8.1  4.7 - 13.4 | 96.1  90.9 - 98.4 | |
| ≥6 | 50.00  15.7 - 84.3 | 62.60  53.7 - 70.9 | .035 | .381 | 1.34  0.6 - 2.8 | 0.80  0.4 - 1.6 | 7.5  3.8 - 14.5 | 95.3  91.0 - 97.6 | |
| ≥7 | 50.00  15.7 - 84.3 | 74.81  66.5 - 82.0 | .092 | .266 | 1.98  0.9 - 4.2 | 0.67  0.3 - 1.3 | 10.8  5.4 - 20.5 | 96.1  92.4 - 98.0 | |
| ≥8 | 50.00  15.7 - 84.3 | 81.68  74.0 - 87.9 | .146 | .201 | 2.73  1.2 - 6.0 | 0.61  0.3 - 1.2 | 14.3  7.1 - 26.7 | 96.4  93.0 - 98.2 | |
| ≥9 | 50.00  15.7 - 84.3 | 85.50  78.3 - 91.0 | .189 | .165 | 3.45  1.5 - 7.7 | 0.58  0.3 - 1.2 | 17.4  8.6 - 32.1 | 96.6  93.3 - 98.3 | |
| ≥10 | 25.00  3.2 - 65.1 | 90.84  84.5 - 95.2 | .117 | .101 | 2.73  0.7 - 10.2 | 0.83  0.6 - 1.2 | 14.3  4.3 - 38.3 | 95.2  93.0 - 96.7 | |
| ≥11 | 25.00  3.2 - 65.1 | 94.66  89.3 - 97.8 | .186 | .065 | 4.68  1.2 - 19.0 | 0.79  0.5 - 1.2 | 22.2  6.6 - 53.7 | 95.4  93.3 - 96.9 | |

Level = Test positive rate (percentage of cases scoring positive)

DLR = Diagnostic likelihood ratio

DLR+ = Ratio between the probability of a positive test result given the presence of the disease and the probability of a positive test result given the absence of the disease

DLR- = Ratio between the probability of a negative test result given the presence of the disease and the probability of a negative test result given the absence of the disease

PPV= Positive predictive value (probability that the disease is present when the test is positive).

NPV = Negative predictive value (probability that the disease is not present when the test is negative).
