# Supplementary material for: Diagnostic efficiency and validity of the DSM-oriented Child Behavior Checklist and Youth Self-Report scales in a clinical sample of Swedish youth
Source: PLoS One. 2021 Jul 22;16(7):e0254953. doi: 10.1371/journal.pone.0254953 (PMC8297893; doi:10.1371/journal.pone.0254953)
Supplement: Attachment — Submitted filename: ASEBA.Halmstad.responses.to.reviewers.docx [file pone.0254953.s003.docx]

**Diagnostic efficiency and validity of the DSM-oriented Child Behavior Checklist and Youth Self-Report scales in a clinical sample of Swedish youth**

**Authors´responses to reviews**

**Editor**

I was hoping to receive a second review of your work, but, unfortunately, it has not been received. I thank the reviewer for their attention to the manuscript. You will see that the reviewer noted a number of clarifications for your work. I concur with the reviewer that the work is done well. The supplementary material, particularly the tables showing the full set of AUC values across scale cutoff values was very helpful. However, I have some additional queries and comments that are intended to help move the work along.

**Authors‘ response**

Thank you.

**Editor**

The motivation for the work on the use of the DSM oriented scale (DOS) scores and diagnostic specificity is clear.

However, there could be some additional details about the motivation to rely on youth and maternal reports, rather than other selection of informants.

**Authors‘ response**

Unfortunately, we only had access to youth and maternal reports. However, we acknowledge that it is a strength to add an additional informant such as father or teacher.

**Editor**

In the initial paragraph of the Methods, you note: “The observation time that yielded new diagnostic information was 1.2 (SD 0.6) years with a range of 0.1–3.1 years.” It is not clear, at this point in the manuscript, what this information is about. Is this about using the LEAD process? If so, this should be integrated into the description.

**Authors‘ response**

Thank you. We have moved this sentence to the measures and procedures section where the LEAD process is described.

**Editor**

Table 2 presents DOS between youth with and without diagnoses and between male and female youth. However, the comparisons of male and female youth are not motivated in the introduction or commented on in the Discussion. You have a choice in how to handle the inclusion/exclusion of these analyses. Please justify your decision; if these are retained be sure to justify their inclusion in the manuscript.

**Authors‘ response**

Thank you.

We have added a sentence in the aim section

„by comparing diagnosis-specific DOS scores between children with and without the diagnosis-specific disorder“.

and

„Our aim was also to examine potential gender differences in mean scores as discovered in our previous papers using same data {Ivarsson et al., 2017, #2921}{Jarbin et al., 2020, #5849}.“

We have also added a paragraph in the discussion

“The results also revealed that boys scored significantly higher on the CBCL ADHD. We did not find any gender differences in other CBCL DOS. Parents ratings for depression and anxiety were similar across gender while girls´ ratings were higher, which is in line with our findings from the Mood and Feelings Questionnaire (MFQ) {Jarbin et al., 2020, #5849} and Screen for Child Anxiety Related Emotional Disorders {Ivarsson et al., 2017, #2921}. Our MFQ study also showed that parents and girls´ report correlated highly. However, the girls scored consequently higher, suggesting that girls express affective symptoms more markedly {Jarbin et al., 2020, #5849}. “

**Editor**

Tables 2 & 3 show associations between DOS scores and diagnoses as mean differences and AUC, respectively. These appear to be reparametrized estimates of the same quantity. Please describe how these are different.

**Authors‘ response**

Thank you. We acknowledge that a part of table 2 (M and SD of diagnosis-specific disorder present/absent) is a reparameterization of table 3. However, we consider both tables to be valuable for our readers (clinicians and researchers). Table 2 added value is to compare M and SDs in this sample to clinical or community samples. Table 3 added value is to present AUCs and the most efficient cutoffs. Please, let us know if you need further elaboration or if changes are suggested.

**Editor**

Moreover, it was not clear whether the models estimated in Table 4 included only a single predictor in each model. If so, then these ORs would be a third representation of the same information. Please clarify whether the models in Table 4 include one or more predictors in the same model.

**Authors‘ response**

Thank you for noting that. We used multivariate logistic regression, all the DOS scales were used as independent variables in each model to determine the specific association between disorder (e.g., depression) and diagnosis-specific scale (e.g., CBCL depression). We apologize for this misunderstanding. We have added “multivariate” in the title. We have also added “multivariate” in statistical analysis and in the results section. We acknowledge that the univariate models are representations of the same information. however, we deem it important as it might be important for our readers to compare the ORs between the univariate and multivariate models.

**Editor**

In the models where youth report was added to parent report, in that order, were parent reports only included if they also had youth reports? If not, then the model R^2^ for the initial step would be based on different data than the model with both informants.

**Authors‘ response**

Thank you for noting that. Yes, we made sure to include only parent reports if they also had youth reports.

**Editor**

In the Discussion, some results are described as if there were direct tests of differences in magnitudes of association. However, the analyses, as presently communicated, are only showing whether there were significant or non-significant associations between DOS scores and diagnoses. This language should be addressed.

**Authors‘ response**

**Thank you. Language is changed in Discussion, 2^nd^ paragraph, line 6.**

**Editor**
Please submit your revised manuscript by Jul 08 2021 11:59PM. If you will need more time than this to complete your revisions, please reply to this message or contact the journal office at [plosone@plos.org](mailto:plosone@plos.org). When you're ready to submit your revision, log on to <https://www.editorialmanager.com/pone/> and select the 'Submissions Needing Revision' folder to locate your manuscript file.

**Editor**
Please include the following items when submitting your revised manuscript:

- A rebuttal letter that responds to each point raised by the academic editor and reviewer(s). You should upload this letter as a separate file labeled 'Response to Reviewers'.
- A marked-up copy of your manuscript that highlights changes made to the original version. You should upload this as a separate file labeled 'Revised Manuscript with Track Changes'.
- An unmarked version of your revised paper without tracked changes. You should upload this as a separate file labeled 'Manuscript'.

**Authors‘ response**

(remember

(1) respones
(2) track changes
(3) CLEAN

If you would like to make changes to your financial disclosure, please include your updated statement in your cover letter. Guidelines for resubmitting your figure files are available below the reviewer comments at the end of this letter.

If applicable, we recommend that you deposit your laboratory protocols in protocols.io to enhance the reproducibility of your results. Protocols.io assigns your protocol its own identifier (DOI) so that it can be cited independently in the future. For instructions see: [http://journals.plos.org/plosone/s/submission-guidelines#loc-laboratory-protocols](https://eur02.safelinks.protection.outlook.com/?url=http%3A%2F%2Fjournals.plos.org%2Fplosone%2Fs%2Fsubmission-guidelines%23loc-laboratory-protocols&data=04%7C01%7Cgskarp%40hi.is%7Cd4a3aeb256ac471c46ee08d920384fdd%7C09fa5f0e211846568529677ed8fdbe78%7C0%7C0%7C637576248501802899%7CUnknown%7CTWFpbGZsb3d8eyJWIjoiMC4wLjAwMDAiLCJQIjoiV2luMzIiLCJBTiI6Ik1haWwiLCJXVCI6Mn0%3D%7C1000&sdata=o3ZM9%2FPWfqPjCP%2FZ%2B4YCoYUfsteSzvRz%2FgI5q%2BHGWOg%3D&reserved=0). Additionally, PLOSONE offers an option for publishing peer-reviewed Lab Protocol articles, which describe protocols hosted on protocols.io. Read more information on sharing protocols at [https://plos.org/protocols?utm_medium=editorial-email&utm_source=authorletters&utm_campaign=protocols](https://eur02.safelinks.protection.outlook.com/?url=https%3A%2F%2Fplos.org%2Fprotocols%3Futm_medium%3Deditorial-email%26utm_source%3Dauthorletters%26utm_campaign%3Dprotocols&data=04%7C01%7Cgskarp%40hi.is%7Cd4a3aeb256ac471c46ee08d920384fdd%7C09fa5f0e211846568529677ed8fdbe78%7C0%7C0%7C637576248501802899%7CUnknown%7CTWFpbGZsb3d8eyJWIjoiMC4wLjAwMDAiLCJQIjoiV2luMzIiLCJBTiI6Ik1haWwiLCJXVCI6Mn0%3D%7C1000&sdata=INrCuBPWomr5onPXWNHx08y5IoWj63SCJgg7lbcXh7A%3D&reserved=0).

We look forward to receiving your revised manuscript.

Kind regards,

Thomas M. Olino
Academic Editor
PLOS ONE

Journal Requirements:

When submitting your revision, we need you to address these additional requirements.

1. Please ensure that your manuscript meets PLOSONE's style requirements, including those for file naming. The PLOS ONE style templates can be found at

<https://journals.plos.org/plosone/s/file?id=wjVg/PLOSOne_formatting_sample_main_body.pdf>and

[https://journals.plos.org/plosone/s/file?id=ba62/PLOSOne_formatting_sample_title_authors_affiliations.pdf](about:blank)

2. Please include your tables as part of your main manuscript and remove the individual files. Please note that supplementary tables should remain uploaded as separate "supporting information" files.

**Authors‘ response**

(Remember to add all tables to the Ms.)

3. Please include captions for your Supporting Information files at the end of your manuscript, and update any in-text citations to match accordingly. Please see our Supporting Information guidelines for more information: [http://journals.plos.org/plosone/s/supporting-information](about:blank).

**Editor**

4. We noticed you have some minor occurrence of overlapping text with the following previous publication(s), which needs to be addressed:

- <https://journals.plos.org/plosone/article?id=10.1371%2Fjournal.pone.0230623>
- <https://link.springer.com/article/10.1007%2Fs10578-017-0746-8>

In your revision ensure you cite all your sources (including your own works), and quote or rephrase any duplicated text outside the methods section. Further consideration is dependent on these concerns being addressed.

**Authors‘ response**

Thank you for noting that. We are the co-authors of both papers. We confirm that we only duplicated some parts of the method section.

[Note: HTML markup is below. Please do not edit.]

Reviewers' comments:

Reviewer's Responses to Questions

**Comments to the Author**

1. Is the manuscript technically sound, and do the data support the conclusions?

The manuscript must describe a technically sound piece of scientific research with data that supports the conclusions. Experiments must have been conducted rigorously, with appropriate controls, replication, and sample sizes. The conclusions must be drawn appropriately based on the data presented.

Reviewer #1: Yes

2. Has the statistical analysis been performed appropriately and rigorously?

Reviewer #1: Yes

3. Have the authors made all data underlying the findings in their manuscript fully available?

The [PLOS Data policy](https://eur02.safelinks.protection.outlook.com/?url=http%3A%2F%2Fwww.plosone.org%2Fstatic%2Fpolicies.action%23sharing&data=04%7C01%7Cgskarp%40hi.is%7Cd4a3aeb256ac471c46ee08d920384fdd%7C09fa5f0e211846568529677ed8fdbe78%7C0%7C0%7C637576248501812890%7CUnknown%7CTWFpbGZsb3d8eyJWIjoiMC4wLjAwMDAiLCJQIjoiV2luMzIiLCJBTiI6Ik1haWwiLCJXVCI6Mn0%3D%7C1000&sdata=Zs9LpKQrPorECj1hFBB1eAdkSbFBbKQQRRgaHvQjEOM%3D&reserved=0) requires authors to make all data underlying the findings described in their manuscript fully available without restriction, with rare exception (please refer to the Data Availability Statement in the manuscript PDF file). The data should be provided as part of the manuscript or its supporting information, or deposited to a public repository. For example, in addition to summary statistics, the data points behind means, medians and variance measures should be available. If there are restrictions on publicly sharing data—e.g. participant privacy or use of data from a third party—those must be specified.

Reviewer #1: Yes

4. Is the manuscript presented in an intelligible fashion and written in standard English?

PLOS ONE does not copyedit accepted manuscripts, so the language in submitted articles must be clear, correct, and unambiguous. Any typographical or grammatical errors should be corrected at revision, so please note any specific errors here.

Reviewer #1: Yes

5. Review Comments to the Author

Please use the space provided to explain your answers to the questions above. You may also include additional comments for the author, including concerns about dual publication, research ethics, or publication ethics. (Please upload your review as an attachment if it exceeds 20,000 characters)

**R1**

Reviewer #1: This is a study on the clinical properties of CBCL, with an excellent experimental design and an advanced analysis of the collected data. It is probably the most accurate CBCL study to date.
The CBCL is a widely used tool, with very high number of citations on PubMed, but there are not many studies on its clinical properties in relation to scaling according to DSM criteria. In the introduction (second page) the authors report these studies; probably for completeness it is appropriate to add the most recent published in Clin Child Psychol Psychiatry 2020;25:507-519. doi: 10.1177/1359104519895056.

**Authors‘ response**

Thank you so much. We have added the study in our manuscript, when we list the studies that have examined the screening efficiency of the DOS depression, anxiety, ADHD, ODD, and CD. Great paper and interesting to read about the CABI.

**R1**
The clinical analysis is particularly accurate according to the criteria of the Longitudinal Expert All Data (LEAD) procedure, even if, considering the variability over time of the clinical picture in children and adolescents, it is questionable whether a final evaluation at an average distance of many months may sometimes not exactly correspond to the clinical situation at the time of administration of the CBCL

**Authors‘ response**

Thank you. We acknowledge, that LEAD diagnoses are not without problems when data over time is used. However, we want to cite the following paper (by the same research group): <http://dx.doi.org/10.1080/08039488.2016.1276622>. In this paper further description on our LEAD method can be accessed. the LEAD diagnoses were based on KSADS data and a minimum of three visits after the KSADS or of significant new information. KSADS diagnoses (at the time of intake) were compared to LEAD diagnoses. The results showed excellent agreement for most diagnoses. For instance, any depression kappa = 0.91, any anxiety disorders kappa = 0.94, any ADHD kappa 0.80. Among these diagnoses, the most notable information arriving after the KSADS-PL was information in rating scales and personal communication from teachers supporting a diagnosis of ADHD. Parents and patients were, at times, reducing their initial description of ADHD and functional impact but agreed later on when school had presented more support and they also were less reluctant of receiving the diagnosis. As ADHD has a chronic fluctuating course, we believe that the later additional information was valuable and correct. There were very few changes of affective diagnosis but in a rare case, the depression was covered by a panic disorder but elicited later on and clearly with an onset before the time of the CBCL/YSR.

**R1**
In the "Diagnostic efficiency" section there is a repetition relating to YSR ODD and OCD.

**Authors‘ response**

Thank you so much. We have changed accordingly by deleting the second sentence about ODD and OCD.

**R1**
In the “Concurrent and discriminant validity” section, please check what is written in relation to the YSR (last lines) in relation to the data in table 4.

**Authors‘ response**

Thank you again. We have modified the sentences according to the results in table 4.

**R1**
As for the incremental validity, I wonder if adding CBCL to YSR and vice versa can increase the accuracy of that of the two which is already more accurate.

Authors‘ response

Again, thank you. We understand that if we add CBCL DOS (e.g., depression) to YSR DOS (e.g., depression) the accuracy of predicting LEAD depression increases and vice versa. Yes, that´s exactly what happens :) if the delta (Δ) (i.e., increase in χ^2^) is significant than adding CBCL to YSR and vice versa increases tha accuracy. Except for adding YSR ODD to CBCL ODD. Adding CBCL CD to YSR CD, and Adding YSR OCD to CBCL OCD.

**R1**
Some minor corrections in the tables.
Table 2 gender t-test YSR ODD -9.360 (probably wrong)

**Authors‘ response**

Wonderful, thank you so much for your sharp eyes. The correct t is 0.936. We missed one decimal.

**R1**
Decimal separator: sometimes is comma instead of dot.

**Authors‘ response**

Thank you and apologies. We have changed from comma to dot accordingly.

6. PLOS authors have the option to publish the peer review history of their article ([what does this mean?](https://eur02.safelinks.protection.outlook.com/?url=https%3A%2F%2Fjournals.plos.org%2Fplosone%2Fs%2Feditorial-and-peer-review-process%23loc-peer-review-history&data=04%7C01%7Cgskarp%40hi.is%7Cd4a3aeb256ac471c46ee08d920384fdd%7C09fa5f0e211846568529677ed8fdbe78%7C0%7C0%7C637576248501812890%7CUnknown%7CTWFpbGZsb3d8eyJWIjoiMC4wLjAwMDAiLCJQIjoiV2luMzIiLCJBTiI6Ik1haWwiLCJXVCI6Mn0%3D%7C1000&sdata=WS%2F1ucW3OaZrqLS3bvcqpOGTAZ0gQQd5Z%2B9I%2BzdzoY0%3D&reserved=0)). If published, this will include your full peer review and any attached files.


If you choose “no”, your identity will remain anonymous but your review may still be made public.


**Do you want your identity to be public for this peer review?** For information about this choice, including consent withdrawal, please see our [Privacy Policy](https://eur02.safelinks.protection.outlook.com/?url=https%3A%2F%2Fwww.plos.org%2Fprivacy-policy&data=04%7C01%7Cgskarp%40hi.is%7Cd4a3aeb256ac471c46ee08d920384fdd%7C09fa5f0e211846568529677ed8fdbe78%7C0%7C0%7C637576248501822887%7CUnknown%7CTWFpbGZsb3d8eyJWIjoiMC4wLjAwMDAiLCJQIjoiV2luMzIiLCJBTiI6Ik1haWwiLCJXVCI6Mn0%3D%7C1000&sdata=e2TR0ok9soEfeawS8UUDycsqwrfvdr1GkEcvMWU4uLs%3D&reserved=0).

Reviewer #1: No

[NOTE: If reviewer comments were submitted as an attachment file, they will be attached to this email and accessible via the submission site. Please log into your account, locate the manuscript record, and check for the action link "View Attachments". If this link does not appear, there are no attachment files.]

While revising your submission, please upload your figure files to the Preflight Analysis and Conversion Engine (PACE) digital diagnostic tool, [https://pacev2.apexcovantage.com/](https://eur02.safelinks.protection.outlook.com/?url=https%3A%2F%2Fpacev2.apexcovantage.com%2F&data=04%7C01%7Cgskarp%40hi.is%7Cd4a3aeb256ac471c46ee08d920384fdd%7C09fa5f0e211846568529677ed8fdbe78%7C0%7C0%7C637576248501822887%7CUnknown%7CTWFpbGZsb3d8eyJWIjoiMC4wLjAwMDAiLCJQIjoiV2luMzIiLCJBTiI6Ik1haWwiLCJXVCI6Mn0%3D%7C1000&sdata=PeF7lrY6W26ELC3lRxZ199e2iV%2BIuXSzNQsCcyF1KzE%3D&reserved=0). PACE helps ensure that figures meet PLOS requirements. To use PACE, you must first register as a user. Registration is free. Then, login and navigate to the UPLOAD tab, where you will find detailed instructions on how to use the tool. If you encounter any issues or have any questions when using PACE, please email PLOSat [figures@plos.org](mailto:figures@plos.org). Please note that Supporting Information files do not need this step.
